# Burden of Respiratory Syncytial Virus Disease in Adults with Asthma and Chronic Obstructive Pulmonary Disease: A Systematic Literature Review

**DOI:** 10.1007/s11882-025-01194-w

**Published:** 2025-02-25

**Authors:** Yolanda Penders, Guy Brusselle, Ann R. Falsey, Gernot Rohde, Estefania Betancur, Maria Elena Guardado, Juan Luis Ramirez Agudelo, Pouya Saeedi, Lauriane Harrington, Jean-Philippe Michaud

**Affiliations:** 1 GSK, Av. Fleming 20, Wavre, 1300 Belgium; 2 Department of Respiratory Medicine, Ghent University Hospital, Ghent, 9000 Belgium; 3 University of Rochester School of Medicine, Rochester, 14642 NY USA; 4 Department of Respiratory Medicine, Medical Clinic I, Goethe University Frankfurt, University Hospital, 60590 Frankfurt/Main, Germany; 5 P95, Leuven, 3000 Belgium

**Keywords:** Adults, Asthma, Chronic obstructive pulmonary disease, Complications, Hospitalization, Respiratory viral infection

## Abstract

**Purpose of Review:**

Accumulating data indicate that asthma and chronic obstructive pulmonary disease (COPD) increase the risk of severe respiratory syncytial virus (RSV) infection. This systematic literature review assessed the burden of RSV disease among adults ≥ 18 years with asthma or COPD.

**Recent Findings:**

Data on the prevalence of asthma or COPD among RSV-infected adults, RSV-related hospitalizations, complications, and mortality were collected from studies published between January 1, 2000 and November 28, 2023 in PubMed, Embase, and grey literature. All extracted data were analyzed descriptively. Pooled estimates of asthma or COPD prevalence among RSV-infected adults were calculated from generalized linear mixed effects model meta-analyses. Forty studies were included. The prevalence of asthma and COPD among RSV-infected adults was high, especially in inpatient settings with pooled estimates (95% confidence interval) of 19.3% (15.0–24.6) for asthma and 30.8% (26.1–36.0) for COPD. Adults with asthma or COPD were more likely to be hospitalized following RSV infection than those without these conditions. The incidence rate ratios of hospitalization were 2.0–3.6 (crude) and 6.7–8.2 (adjusted) for asthma and 3.2–13.4 (crude) and 9.6–9.7 (adjusted) for COPD. The most frequently reported RSV-related complications were exacerbation of asthma (up to 64.9%) and COPD (up to ≥ 83.0%). In-hospital case fatality rates were 2.6–4.3% (asthma) and 2.8–17.8% (COPD).

**Summary:**

These comprehensive data showing a high RSV disease burden in adults with asthma or COPD can be used to inform policy decisions around RSV vaccines and improve preventive care in this high-risk population.

**Graphical Abstract:**

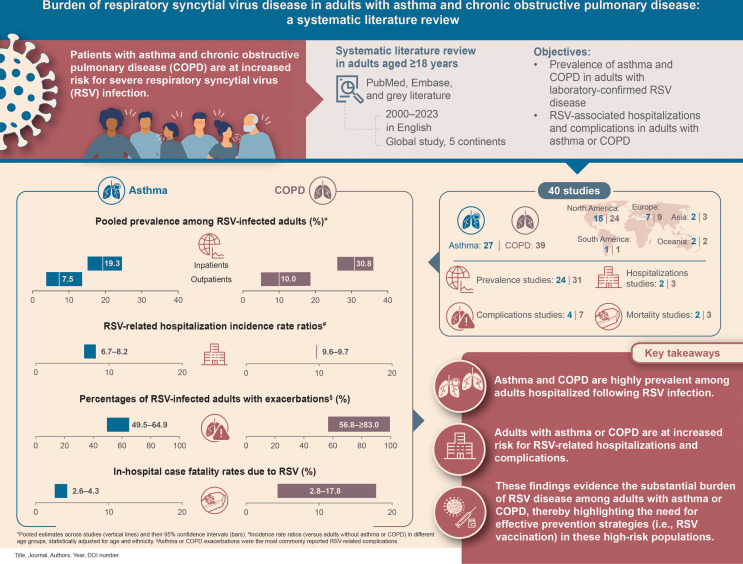

**Supplementary Information:**

The online version contains supplementary material available at 10.1007/s11882-025-01194-w.

## Plain Language Summary

**What is the context?**

• Respiratory syncytial virus (RSV) is a virus that infects the lungs and airways. RSV typically causes mild respiratory illnesses in healthy people. However, RSV disease may be severe and sometimes fatal in people with chronic medical conditions like asthma or chronic obstructive pulmonary disease (COPD).

• We analyzed data from 40 scientific studies to find out how RSV disease impacts adults aged 18 years or older with asthma or COPD.

**What is new?**

• The analysis of included studies showed that, out of 100 adults hospitalized for RSV infection, an average of 19 had asthma, and an average of 31 had COPD.

• Up to 8 times more adults with asthma and 13 times more of those with COPD were hospitalized due to an RSV infection compared to people without these lung diseases.

• According to 3 studies, adults with asthma or COPD frequently suffered a worsening of their lung disease after RSV infection. Some adults became critically ill and required intensive care to treat severe RSV symptoms (including mechanical ventilation to help them breathe).

• Furthermore, among the hospitalized people infected with RSV, 3 to 4 out of 100 adults with asthma and 3 to 18 out of 100 adults with COPD eventually died during an acute RSV infection.

**What is the impact?**

• Adults with asthma or COPD are at high risk of developing severe RSV symptoms requiring hospital care or even resulting in death.

• Adults with asthma or COPD are likely to benefit from newly available RSV vaccines to protect them against severe RSV infection and associated medical problems.

**Supplementary Information:**

The online version contains supplementary material available at 10.1007/s11882-025-01194-w.

## Introduction

Respiratory syncytial virus (RSV) is one of the most common etiologic agents of acute respiratory illness worldwide [1]. The clinical spectrum of RSV disease ranges from mild cold-like symptoms to severe lower respiratory tract disease potentially causing hospitalization, intensive care unit (ICU) admission, and/or death [2, 3]. The burden of RSV disease has been primarily recognized in infants and young children [4, 5]. More recently, its contribution to morbidity and mortality among adults at increased risk of developing severe RSV infection, such as older adults and those with certain comorbidities, has been progressively documented through accumulating epidemiological data [6–8]. For instance, a meta-analysis estimated that in 2019, RSV accounted for 5.2 million cases of acute respiratory infection (ARI), 470,000 hospitalizations, and 33,000 in-hospital deaths among adults ≥ 60 years of age (YOA) in high-income countries [8]. Recent changes in RSV testing practices, including more frequent molecular screening for viral pathogens in adults since the coronavirus disease 2019 (COVID-19) pandemic, could have also contributed to a better characterization of RSV disease burden among high-risk adults [9]. The growing recognition of RSV disease burden accelerated vaccine development to meet the medical need for RSV prophylactic interventions in target populations [10]. The currently available vaccines for the prevention of lower respiratory tract disease caused by RSV (RSV-LRTD) in adults—adjuvanted RSVPreF3 (*Arexvy*, GSK), RSVpreF (*Abrysvo*, Pfizer Inc.), and mRNA-1345 (*mRESVIA*, Moderna)—are approved for use in people ≥ 60 YOA [11–13], with an expanded indication in the United States (US) to adults at increased risk for RSV-LRTD for 2 of these vaccines (adjuvanted RSVPreF3: adults 50–59 YOA; RSVpreF: adults 18–59 YOA) [14, 15]. Of note, the situation is dynamic and the indications for these vaccines can be expected to change in the near future.

Besides the effects of aging and associated immunosenescence, there is a growing body of evidence documenting the increased severity of RSV infection in adult groups with different comorbidities, particularly those with chronic respiratory diseases such as asthma and chronic obstructive pulmonary disease (COPD) [6–8, 16, 17]. Several studies have identified RSV as a major viral contributor to acute asthma and COPD exacerbations based on detection from respiratory samples (e.g., nasal aspirate/swab, oropharyngeal and nasopharyngeal lavage/swab, spontaneously secreted and induced sputum, bronchoalveolar lavage fluid) [7, 18–22]. In addition, other respiratory conditions (e.g., pneumonia and respiratory failure) may develop as severe complications of lower respiratory tract infection in hospitalized RSV-infected adults [6, 23, 24]. Beyond these acute respiratory manifestations, RSV infection may lead to severe long-term complications in people with risk factors (e.g., cardiovascular and respiratory conditions), including an accelerated decline in lung function, altered physical functional status with associated impact on dependency and quality of life, and increased mortality [18, 25–27]. The burden and severity of RSV disease were found to be comparable or even higher than other viral respiratory infections (e.g., influenza, COVID-19) in hospitalized adults [28, 29], including those with asthma or COPD [25, 30]. Despite this clinical evidence demonstrating the public health importance of RSV, it is necessary to increase clinicians’ knowledge of the symptoms and risk factors predictive of severe RSV infection in adults [31].

Recent systematic literature reviews (SLRs) have provided various estimates of RSV burden in terms of disease prevalence, RSV-related hospitalizations, healthcare utilization, and mortality among adults with comorbidities [6, 7, 32]. While some of these studies reported stratified data in asthma or COPD groups [6, 7], they did not extensively focus on these respiratory diseases and were generally limited in terms of publication period covered (i.e., not including data published before 2012 [6] or after 2019 [7]). With the recent approvals of RSV vaccines, robust data and an accurate estimation of RSV disease burden in adults with asthma or COPD are critical in building awareness of risk factors, informing risk-benefit assessments to support policy decisions around vaccination recommendations in adults at increased risk, and fostering discussions between patients and physicians regarding RSV vaccination.

We performed an SLR to provide a comprehensive, up-to-date overview of RSV disease burden in adults ≥ 18 YOA with asthma or COPD and to identify potential knowledge gaps, based on an extensive literature search covering the 2000–2023 publication period. The objectives of this study were to assess the burden of RSV in adults with asthma or COPD through different outcomes, including (1) the prevalence of these respiratory conditions among patients with laboratory-confirmed RSV infection, (2) RSV-related hospitalization rates and risk, (3) complications, and (4) mortality in adults with asthma or COPD.

## Methods

### Eligibility Criteria

This SLR was prepared following the Preferred Reporting Items for Systematic reviews and Meta-Analyses (PRISMA) guidelines [33]. It included studies reporting on the burden of RSV disease in adults ≥ 18 YOA with asthma or COPD in high-income countries (according to the World Bank classification [34]).

Only English-language studies were included. Populations of interest were inpatient (hospitalized for at least 1 night, also including patients admitted to the ICU) and/or outpatient adults (non-hospitalized, also including medically attended outpatients who visited their general practitioner or the emergency department) with RSV infection confirmed by laboratory testing (mostly through polymerase chain reaction [PCR]). This SLR used stringent laboratory-confirmed RSV case definition (compared with other diagnosis methods such as International Classification of Diseases [ICD] codes) to ensure robust and consistent identification of RSV-positive individuals. Only studies reporting on outcomes of interest in patients with asthma or COPD were included. Studies focusing on a non-relevant (e.g., immunosuppressed patients) or non-representative population (e.g., convenience sample, studies where only influenza-negative patients were tested for other pathogens) were excluded. Studies that did not specify the laboratory method used for confirming RSV infection during primary diagnostic or only relied on an insufficiently sensitive method (e.g., rapid antigen testing), had a sample size *n* < 20, or did not provide sufficient details to extract relevant data were excluded as well (Online Resource 1).

## Literature Search Strategy

A systematic literature search was conducted in PubMed and Embase databases, to identify studies published between January 1, 2000 and November 28, 2023 (date of the literature search) describing the impact of RSV on adults with asthma or COPD, including risk factors and complications (Online Resource 2). Review articles identified during the search and covering the objectives of the present study were discarded, but their reference lists were screened to retrieve additional relevant articles published throughout the period of interest.

Additionally, a comprehensive search of the most up-to-date grey literature (conference abstracts) published between December 5, 2021 and December 7, 2023 (date of grey literature search) was conducted (**Online Resource 3**).

## Literature Screening and Data Extraction

After duplicate removal, all identified records underwent title and abstract screening by 2 independent epidemiologists, and any record evaluated to be relevant to the objectives of this SLR by at least 1 reviewer was considered eligible for full-text screening.

Full-text screening was done by 3 independent epidemiologists. A fourth epidemiologist independently screened 30% of full-text articles, to ensure that no relevant articles were excluded. Any discrepancy between the reviewers was discussed to achieve alignment.

Data extraction was led by the same 3 independent epidemiologists already involved in full-text screening. During this phase, articles found to present similar results from overlapping datasets were excluded to keep only the article with the most complete dataset (or the most recently published one if datasets were identical). Articles presenting different results from overlapping datasets (i.e., reporting on different outcomes or group stratifications) were retained for data extraction. Any doubt during data extraction was discussed with a fourth epidemiologist, who also reviewed 10% of the extracted data in detail. Data were extracted in a summary spreadsheet, using the following categories:

- publication details: authors, year of publication, title;
- general study information: study objectives and design, country/region and period of data collection, RSV detection method (e.g., PCR, viral culture);
- characteristics of enrolled population: patient settings (inpatient, outpatient, or mixed [inpatient/outpatient]), sample size, gender, age, comorbidities;
- outcome of interest (stratified by age, comorbidity of interest [asthma or COPD], and patient setting if available): prevalence of asthma or COPD among adults with RSV (i.e., percentage of RSV-infected patients with asthma or COPD), RSV-related hospitalization rates (i.e., number of hospitalizations attributable to RSV reported per unit of person-time or per population) among adults with asthma or COPD, risk of RSV-related hospitalization among adults with asthma or COPD relative to those without these comorbidities (expressed as incidence rate ratio [IRR] or odds ratio [OR], crude or adjusted, with accompanying *p* value and statistical methods for adjustment), percentage of RSV-infected patients with asthma or COPD experiencing RSV-related complications, and case fatality rates associated with RSV among adults with asthma or COPD (i.e., percentage of adults with RSV and asthma or COPD who died following RSV infection, also including place, time frame, and cause of death) (Online Resource 1).

### Critical Appraisal

The methodological quality of each study was assessed using the critical appraisal checklists developed by the Joanna Briggs Institute (JBI) at the University of Adelaide, Australia [35]. JBI tools determine the extent to which a study was appropriately designed to demonstrate its own objectives and if the study has addressed the possibility of bias in its design, conduct, and analysis. The checklists used to evaluate study methodological quality in the context of this SLR included between 8 and 13 items. Each item of the checklist could be answered by “yes” (criterion met), “no” (criterion unmet), “unclear”, or “not applicable”. The assessment of study methodological quality was based on a non-validated scoring system considering the number of items with a “no” or “unclear” answer. A distinction was made between studies of “sufficient” (< 4 items with no/unclear answer) and “poor” quality (≥ 4 items with no/unclear answer).

### Statistical Analyses

All extracted data were presented descriptively for each comorbidity group (asthma or COPD) and each outcome. Pooled estimates of the prevalence of asthma or COPD among adults with RSV were calculated from individual percentages documented in different studies. Pooled estimates were calculated based on generalized linear mixed effects model meta-analyses incorporating a logit link function and using the maximum likelihood method. Individual percentages stratified by age group were included separately in the meta-analyses instead of collapsing all age groups per study, as this procedure did not affect the pooled estimates. Pooled estimates were expressed in percentages and calculated for each comorbidity group, including the overall prevalence (descriptive estimation across all included studies) and prevalence stratified by patient setting (inpatient or outpatient). Pooled estimates for the mixed (inpatient/outpatient) population were included for descriptive and sensitivity analysis purposes, to validate subgroup estimates and provide context for broader interpretations. The sensitivity analysis was limited to studies conducted in the US (the country with the highest number of eligible studies for pooling). Confidence intervals (CI) associated with individual percentages were adjusted using the binomial exact (Clopper-Pearson) method. Between-study data heterogeneity was quantified with *I*² statistic and statistically evaluated using Cochran’s *Q*-test. Prediction intervals were calculated and provided alongside pooled estimates. No data were pooled for the other RSV burden estimates included in the SLR due to an insufficient number of studies available and considerable heterogeneity in the outcomes. Data were analyzed in R version 4.3.2 (R Foundation for Statistical Computing, Vienna, Austria).

## Results

### Systematic Literature Review

**Fig. 1:**
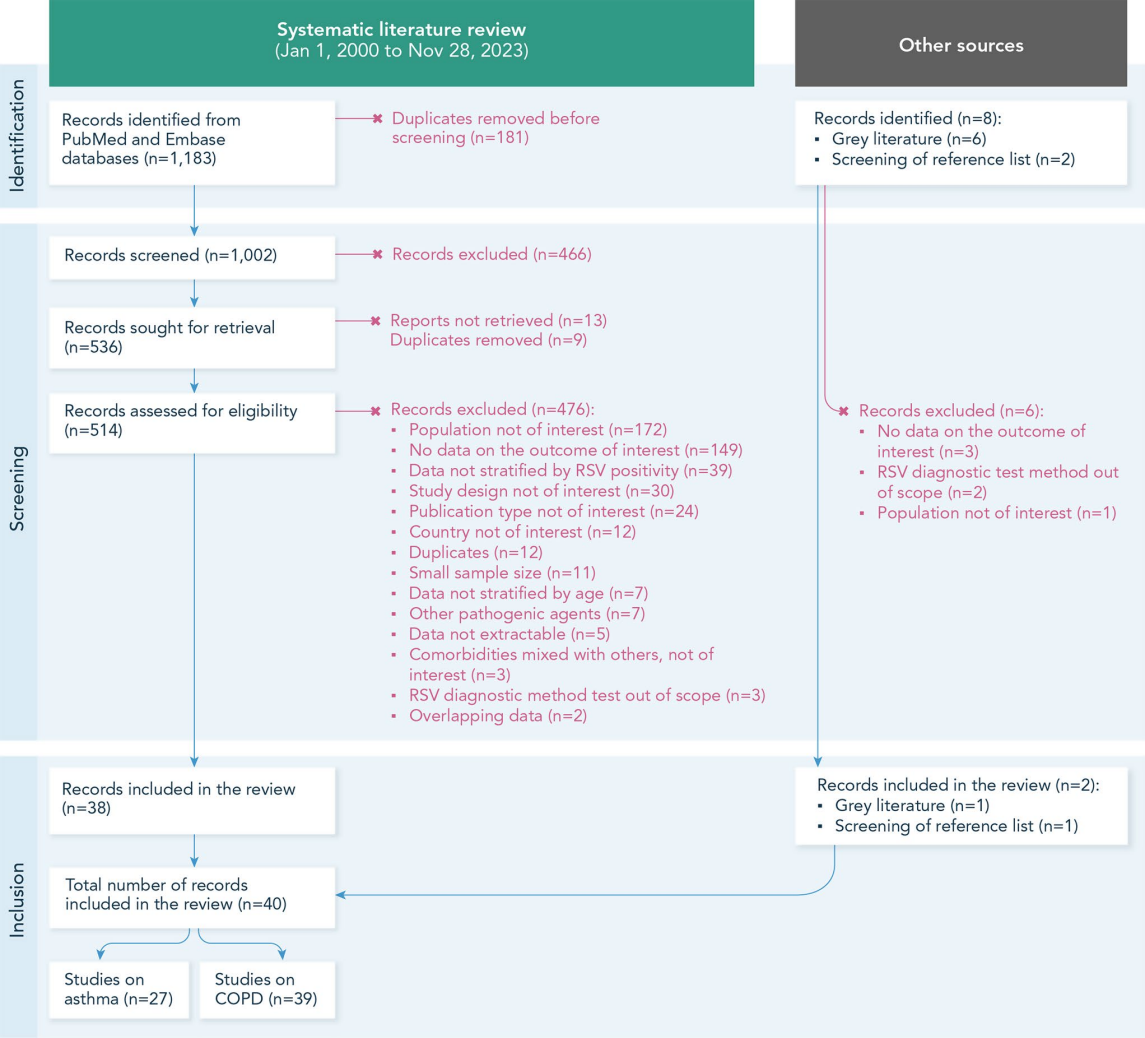
PRISMA flow chart of systematic literature search. Note: The term “screening of reference lists” refers to screening of reference lists of review articles identified during the literature search but not included in this systematic review (see also Methods section). Abbreviations: COPD, chronic obstructive pulmonary disease; n, number of records at each step; RSV, respiratory syncytial virus

This SLR included a total of 40 studies: 38 from database literature [25, 26, 36–71], 1 from grey literature [72], and 1 from screening of reference lists of identified review articles [73] (Fig. 1). Of these, 27 studies included asthma as a comorbidity [25, 26, 36, 39, 41–43, 45–54, 56, 60, 62–65, 67, 69, 71, 73] and 39 included COPD [25, 26, 36–66, 68–73] (Online Resource 4).

### Critical Appraisal

Thirty-five of the 40 studies (87.5%) included in this SLR were rated as having a sufficient methodological quality, according to the JBI appraisal tools (Online Resource 4). Of the 5 studies assessed as poor in terms of methodological quality, 4 were retrospective cohort studies [37–40] and 1 was a prospective cohort study [53]. Of note, the inclusion of these studies in the dataset did not impact the conclusions of the present SLR. The main quality issues noted during evaluation were related to a lack of a clearly defined strategy to address and mitigate confounding factors (no/unclear answer for 4 studies), the low validity and reliability of methods used for measuring the outcomes of interest (no/unclear answer for 4 studies), and the low relevance of statistical analysis performed (no/unclear answer for 3 studies).

### Asthma

#### Characteristics of Included Studies

Of the 27 studies focusing on adults with RSV and asthma, most were conducted in North America (*n* = 15), followed by Europe (*n* = 7), Asia (*n* = 2), Oceania (*n* = 2), and South America (*n* = 1). Most studies (*n* = 22) analyzed inpatient data, followed by mixed inpatient/outpatient (*n* = 3), and outpatient settings (*n* = 3). One study reported on separate inpatient and outpatient populations and was therefore included in both the inpatient and outpatient study counts [45]. All studies were observational and predominantly relied on prospective or retrospective cohort designs (Online Resource 4).

#### Prevalence of Asthma among Adults with RSV

**Fig. 2:**
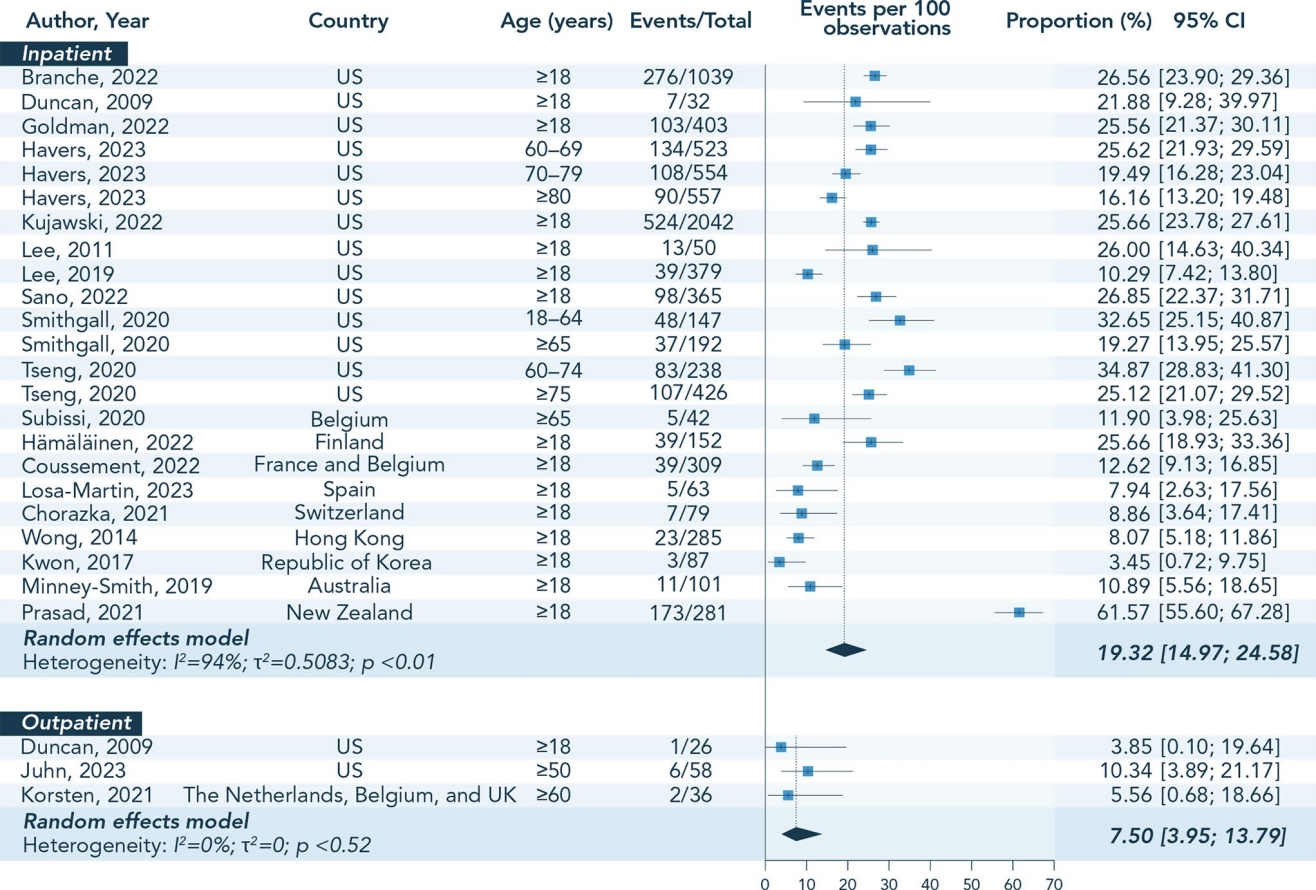
Individual percentages and pooled estimates of asthma prevalence among adults with RSV, stratified by age group and patient setting. Abbreviations: CI, confidence interval; RSV, respiratory syncytial virus; UK, United Kingdom; US, United States

Three studies were excluded from the analysis of pooled prevalence estimate because of overlapping data [25, 26, 36] (Online Resource 4). Based on 24 studies (covering 14 countries), the overall prevalence of asthma among RSV-infected adults ≥ 18 YOA ranged between 2.2% [73] and 61.6% [62], with approximately 60% (14/24) of studies reporting percentages in the range of 10.0–30.0%. Asthma was generally more prevalent in adults ≥ 60 YOA (range: 5.6–28.6%) than in the overall population ≥ 18 YOA. Based on the meta-analysis using the random effect model, the overall pooled estimate of asthma prevalence across all studies was 17.7% (95% CI: 13.7–22.7), with a high level of between-study heterogeneity (*I*²=93%). A higher prevalence of asthma was generally observed in inpatient (range: 3.5–61.6%; pooled estimate and 95% CI: 19.3% [15.0–24.6]; *I*²=94%) versus outpatient settings (range: 3.9–10.3%; pooled estimate and 95% CI: 7.5% [4.0–13.8]; *I*²=0%) (Fig. 2). The pooled estimate of asthma prevalence in mixed inpatient/outpatient settings was 12.0% (95% CI: 9.0–15.9; *I*²=43%).

#### RSV-Related Hospitalization Rates and Risk among Adults with Asthma

Two population-based surveillance studies reported on RSV-related hospitalization rates (per 100,000 persons) and risk among adults (≥ 18 YOA) with ARI symptoms and asthma over 3 RSV seasons [41, 62] (Table 1). In the US-based study by Branche et al. [41], the incidence of RSV-associated hospitalizations in adults with asthma was estimated by dividing the number of hospitalized RSV-infected adults by the market-share-adjusted US Census population estimate and using condition-specific denominators. IRRs with 95% CIs were estimated by comparing incidence rates for hospitalized adults with and without each condition. Branche et al. reported on hospitalization rates between 14.7 in adults 18–49 YOA and 369.9 in those ≥ 65 YOA [41]. In New Zealand, Prasad et al. calculated age- and ethnicity-adjusted incidence rates by dividing the number of RSV-associated ARI hospitalizations (singular episodes) by the number of adults with a given underlying medical condition [62]. RSV hospitalization IRRs were calculated by comparing individuals with an underlying condition to those without this condition. CIs for incidence rates and IRRs were based on the Poisson distribution. Prasad et al. reported on hospitalization rates ranging from 13.6 (95% CI: 8.6–18.6) in adults 18–49 YOA to 119.6 (95% CI: 92.1–147.1) in those 65–80 YOA [62]. In both studies, adults with asthma had a significantly higher risk of RSV-related hospitalization compared to those without asthma, and the magnitude of risk generally increased with age. According to Branche et al., the crude IRR of hospitalization in adults with versus without asthma varied from 2.0 (95% CI: 1.0–4.1) in adults 18–49 YOA to 3.6 (95% CI: 2.2–5.8) in those 50–64 YOA [41]. The adjusted IRR values reported by Prasad et al. ranged from 6.7 (95% CI: 4.1–11.0) in adults 18–49 YOA to 8.2 (95% CI: 5.5–12.2) in those 65–80 YOA [62].

**Table 1:** RSV-related hospitalization rates and hospitalization risk among adults with asthma or COPD

Reference (study location)	Patient settings (population description)	Age group	Total sample size	Number of patients with the comorbidity of interest	Hospitalization rate per 100,000 persons (95% CI)	IRR or OR (95% CI)
**Asthma**						
Branche, 2022 [41] (US)	Inpatient (community cohort of adults who sought care for ARI in outpatient clinics or hospital settings)	18–49 YOA	NYC: 91 Rochester: 57	NYC: 143 Rochester: 133	NYC: 15.6 Rochester: 14.7	NYC (crude IRR): 2.0 (1.0–4.1) Rochester (crude IRR): 2.4 (0.7–7.9)
		50–64 YOA	NYC: 147 Rochester: 133		NYC: 110.9 Rochester: 90.2	NYC (crude IRR): 3.6 (2.2–5.8) Rochester (crude IRR): 2.3 (0.7–7.4)
		≥ 65 YOA	NYC: 332 Rochester: 279		NYC: 369.9 Rochester: 261.4	NYC (crude IRR): 2.3 (1.7–3.1) Rochester (crude IRR): 2.5 (0.8–7.9)
Prasad, 2021 [62] (New Zealand)	Inpatient (hospitalized patients, suspected ARI cases, and those who met the World Health Organization SARI case definition)	18–49 YOA	597,167^a^	61,110	13.6 (8.6–18.6)	Adjusted IRR^b^: 6.7 (4.1–11.0)
		50–64 YOA	188,157^a^	26,676	49.8 (36.3–63.3)	Adjusted IRR^b^: 7.6 (4.9–11.6)
		65–80 YOA	98,675^a^	18,603	119.6 (92.1–147.1)	Adjusted IRR^b^: 8.2 (5.5–12.2)
**COPD**						
Branche, 2022 [41] (US)	Inpatient	18–49 YOA	NYC: 91 Rochester: 57	NYC: 126 Rochester: 156	NYC: 46.8 Rochester: 24.9	NYC (crude IRR): 5.6 (1.7–18.1) Rochester (crude IRR): 3.2 (1.0–10.2)
		50–64 YOA	NYC: 147 Rochester: 133		NYC: 210.3 Rochester: 204.8	NYC (crude IRR): 6.3 (3.8–10.6) Rochester (crude IRR): 6.4 (2.0–20.1)
		≥ 65 YOA	NYC: 332 Rochester: 279		NYC: 529.2 Rochester: 1,077.4	NYC (crude IRR): 3.5 (2.6–4.7) Rochester (crude IRR): 13.4 (4.3–42.0)
Duncan, 2009 [45] (US)	Inpatient and outpatient (independently living adults, hospital employees with ARTI, adults with ARTI evaluated in the emergency department and/or admitted to the hospital)	≥ 18 YOA	58	24	NR	Adjusted OR^c^: 4.6 (1.2–17.7); *p* = 0.02
Prasad, 2021 [62] (New Zealand)	Inpatient	50–64 YOA	188,157^a^	15,046	69.6 (49.0–90.8)	Adjusted IRR^b^: 9.6 (6.2–14.8)
		65–80 YOA	98,675^a^	16,606	135.2 (101.8–168.6)	Adjusted IRR^b^: 9.7 (6.3–14.9)

^a^ Sample size corresponds to the total population of the region where the study took place

^b^ IRR statistically adjusted for age and ethnicity

^c^ OR statistically adjusted for log_10_ (viral load + 1), COPD, diabetes mellitus, any cardiac condition (coronary artery disease, congestive heart failure, or both), steroid use, and continuous age (multiple logistic regression)

Abbreviations: CI, confidence interval; COPD, chronic obstructive pulmonary disease; IRR, incidence rate ratio; NR, not reported; NYC, New York City; OR, odds ratio; RSV, respiratory syncytial virus; (S)AR(T)I, (severe) acute respiratory (tract) infection; US, United States; YOA, years of age

#### RSV-Related Complications among Adults with Asthma

Four studies included data on the percentage of hospitalized RSV-infected patients with asthma who experienced complications, including asthma exacerbation [25, 69], need for mechanical ventilation [71], and severe clinical outcomes (including ICU admission, mechanical ventilation, and/or RSV-associated death during hospitalization) [46] (Table 2). Asthma exacerbation occurred in 49.5–64.9% of hospitalized patients ≥ 60 YOA [25, 69]. Among asthmatic adults ≥ 18 YOA, 8.7% required mechanical ventilation and 20.4% experienced severe clinical outcomes [46, 71].

**Table 2:** RSV-related complications among adults with asthma or COPD

Reference (study location)	Patient settings (population description)	Age group	Sample size in RSV population	Type of complication	Prevalence (%) or risk (HR [95% CI])
**Asthma**					
Ackerson, 2019 [25] (US)	Inpatient (hospitalized KPSC members with a positive RSV or influenza A/B test)	≥ 60 YOA	168	Asthma exacerbation	64.9%
Goldman, 2022 [46] (US)	Inpatient (adults hospitalized with laboratory-confirmed RSV infection)	≥ 18 YOA	103	Severe clinical outcomes^a^	20.4%
Tseng, 2020 [69] (US)	Inpatient (hospitalized KPSC members with a positive RSV test)	≥ 60 YOA	190	Asthma exacerbation	49.5%
Wong, 2014 [71] (Hong Kong)	Inpatient (adults hospitalized with laboratory-confirmed RSV infection)	≥ 18 YOA	23	Mechanical ventilation	8.7%
**COPD**					
Ackerson, 2019 [25] (US)	Inpatient	≥ 60 YOA	192	COPD exacerbation	56.8%
Goldman, 2022 [46] (US)	Inpatient	≥ 18 YOA	91	Severe clinical outcomes^a^	26.4%
Mehta, 2013 [59] (US)	Inpatient and outpatient (cohort of adults ≥ 21 YOA at increased risk, hospitalized cohort of adults ≥ 65 YOA with ARI symptoms or cardiopulmonary condition, patients ≥ 40 YOA with physician-diagnosed COPD and past or active smoking)	≥ 21 YOA	42	COPD exacerbation	≥ 83.0%^b^
Mulpuru, 2022 [61] (Canada)	Inpatient (adults hospitalized with ARI)	≥ 50 YOA	145	ICU admission	17.9%
				Mechanical ventilation	9.0%
Stolz, 2019 [66] (Switzerland)	Outpatient (adults with COPD and a smoking history of ≥ 10 pack-years)	> 40 YOA	29	COPD exacerbation	RSV-A: 1.2 (0.2–8.8) RSV-B: 1.9 (0.4–8.9)
Tseng, 2020 [69] (US)	Inpatient	≥ 60 YOA	235	COPD exacerbation	80.4%
Wong, 2014 [71] (Hong Kong)	Inpatient	≥ 18 YOA	71	Mechanical ventilation	21.1%

^a^ Severe clinical outcomes include ICU admission, receipt of mechanical ventilation, and/or RSV-associated death during hospitalization

^b^ Most patients experienced symptoms consistent with acute exacerbation of COPD, with 83% complaining of cough, 62% increased sputum production and dyspnea, and 48% wheezing

Abbreviations: ARI, acute respiratory infection; CI, confidence interval; COPD, chronic obstructive pulmonary disease; HR, hazard ratio; ICU, intensive care unit; KPSC, Kaiser Permanente Southern California; RSV, respiratory syncytial virus; US, United States; YOA, years of age

#### Mortality Associated with RSV among Adults with Asthma

Two studies documented case fatality rates related to RSV infection among hospitalized adults ≥ 18 YOA with asthma [47, 71] (Table 3). These studies quantified case fatality rates of 2.6% (all-place and all-cause mortality during hospitalization and within 30 days after hospital discharge) [47] and 4.3% (in-hospital mortality) [71].

**Table 3:** Mortality associated with RSV among adults with asthma or COPD

Reference (study location)	Patient settings (population description)	Age group	Sample size in RSV population	Case fatality rate	Place, cause, and time of death
**Asthma**					
Hämäläinen, 2022 [47] (Finland)	Inpatient (patients treated due to influenza and RSV)	≥ 18 YOA	39	2.6%	All-place and all-cause mortality during hospitalization and 30 days after hospital discharge
Wong, 2014 [71] (Hong Kong)	Inpatient (adults hospitalized with laboratory-confirmed RSV infection)	≥ 18 YOA	23	4.3%	In-hospital mortality
**COPD**					
Hämäläinen, 2022 [47] (Finland)	Inpatient	≥ 18 YOA	20	10.0%	All-place and all-cause mortality during hospitalization and 30 days after hospital discharge
Mulpuru, 2022 [61] (Canada)	Inpatient (adults hospitalized with ARI)	≥ 60 YOA	145	2.8%	Inpatient mortality 30 days after hospital discharge
Wong, 2014 [71] (Hong Kong)	Inpatient	≥ 18 YOA	73	17.8%	In-hospital mortality

Abbreviations: ARI, acute respiratory infection; COPD, chronic obstructive pulmonary disease; RSV, respiratory syncytial virus; YOA, years of age

### COPD

#### Characteristics of Included Studies

Among the 39 studies involving RSV-infected adults with COPD, the majority occurred in North America (*n* = 24), followed by Europe (*n* = 9), Asia (*n* = 3), Oceania (*n* = 2), and South America (*n* = 1). These studies were mostly conducted in inpatient settings (*n* = 29), followed by mixed inpatient/outpatient settings (*n* = 6), and outpatient settings (*n* = 6). Two studies reported on separate inpatient and outpatient populations and were therefore included in both inpatient and outpatient study counts [45, 70]. With the exception of 1 randomized controlled trial, all studies were observational and most involved a prospective or retrospective cohort design (Online Resource 4).

#### Prevalence of COPD among Adults with RSV

**Fig. 3:**
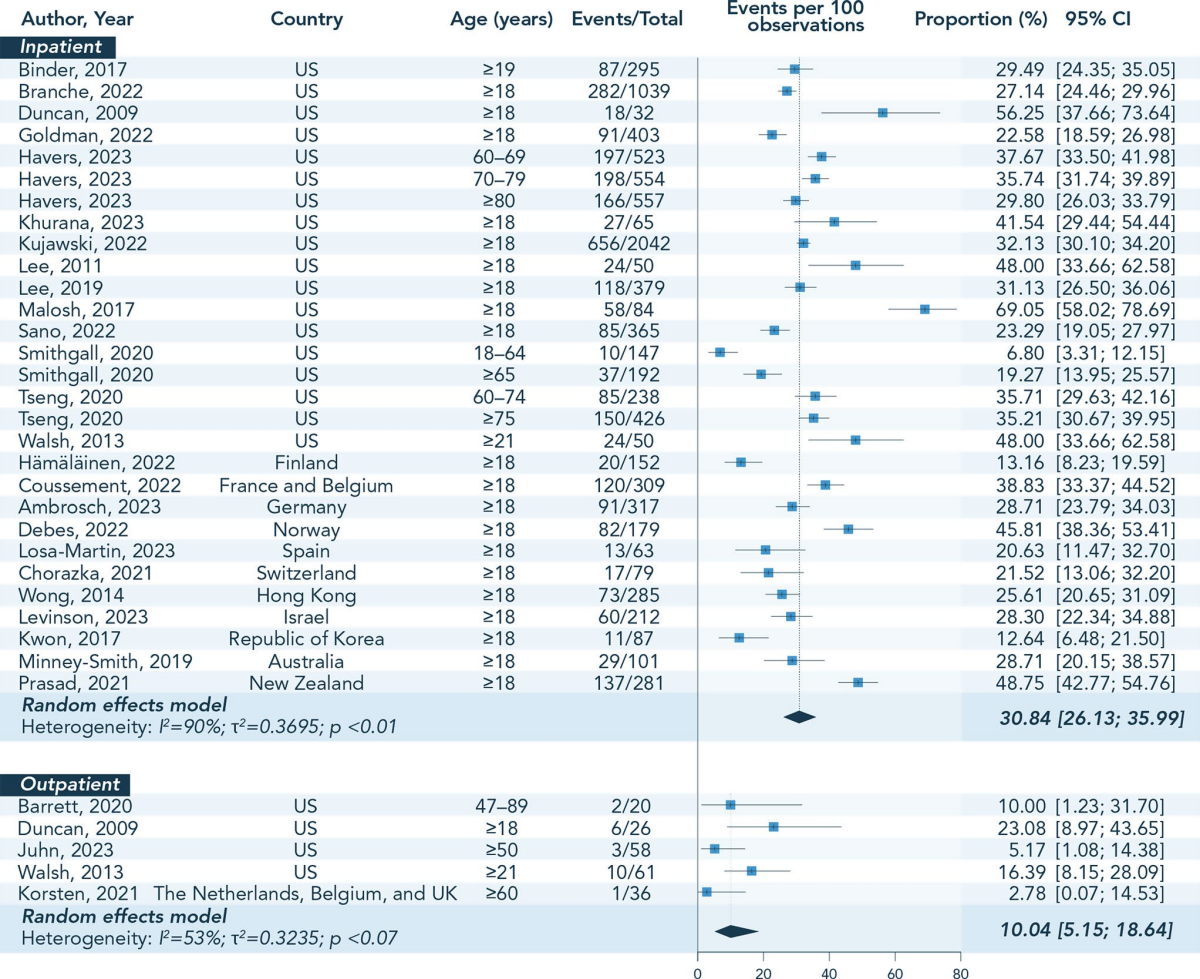
Individual percentages and pooled estimates of COPD prevalence among adults with RSV, stratified by age group and patient setting. Abbreviations: CI, confidence interval; COPD, chronic obstructive pulmonary disease; RSV, respiratory syncytial virus; UK, United Kingdom; US, United States

Of the 36 studies providing data on the prevalence of COPD among adults with RSV, 5 were excluded from pooled estimate analysis because of overlapping data [25, 26, 36, 39, 58] (Online Resource 4). There was a large variation in COPD prevalence across the 31 studies selected for data extraction (covering 17 countries), the lowest percentage being 2.8% [50] and highest 69.1% [57], although 50% (16/31) of the studies provided values in the range of 20.0–40.0%. One study also evidenced an age-related increase in the prevalence of COPD between RSV-infected adults 18–64 YOA (6.8%) and those ≥ 65 YOA (19.3%) [65]. The meta-analysis with random effect model provided an overall pooled estimate of COPD prevalence of 27.7% (95% CI: 22.9–33.0), with a high level of between-study heterogeneity (*I*²=89%). COPD was generally more prevalent in inpatient settings (range: 6.8–69.1%; pooled estimate and 95% CI: 30.8% [26.1–36.0]; *I*²=90%) versus outpatient settings (range: 2.8–23.1%; pooled estimate and 95% CI: 10.0% [5.2–18.6]; *I*²=53%) (Fig. 3). In outpatient settings, one retrospective study reported on COPD prevalence of 10.0% among RSV-infected adults living in long-term care facilities [38], which was within the range (2.8–23.1%) reported by the remaining 4 prospective studies following adults living independently in the community during RSV season [45, 49, 50, 70]. The pooled estimate of COPD prevalence in mixed inpatient/outpatient settings was 12.6% (95% CI: [4.9–28.8]; *I*²=91%).

#### RSV-Related Hospitalization Rates and Risk among Adults with COPD

Three studies reported on the risk of RSV hospitalization among patients with COPD [41, 45, 62], of which 2 also provided data on associated hospitalization rates [41, 62] (Table 1). Highest hospitalization rates (per 100,000 persons) were noted in the older age group (≥ 65 YOA) in both studies. Branche et al. reported on hospitalization rates ranging from 24.9 in adults 18–49 YOA to 1,077.4 in those ≥ 65 YOA [41]. The hospitalization rates in the Prasad et al. study were between 69.6 (95% CI: 49.0–90.8) in adults 50–64 YOA and 135.2 (95% CI: 101.8–168.6) in those 65–80 YOA [62]. All included studies consistently found a significantly higher risk of RSV-related hospitalization among adults with COPD compared to those without COPD. The increased risk of RSV-related hospitalization due to COPD was observed across all age strata, and the magnitude of this increase was independent from age. The crude IRR reported by Branche et al. was between 3.2 (95% CI: 1.0–10.2) in adults 18–49 YOA and 13.4 (95% CI: 4.3–42.0) in those ≥ 65 YOA [41]. Prasad et al. reported on adjusted IRR ranging from 9.6 (95% CI: 6.2–14.8) in adults 50–64 YOA to 9.7 (95% CI: 6.3–14.9) in those ≥ 65 YOA [62]. In contrast to studies in inpatients [41, 62], Duncan et al. analyzed data from both inpatients and outpatients from a single center in the US and over a single RSV season. The ORs of being an inpatient versus outpatient, and of undergoing ventilation versus not undergoing ventilation were modeled using multiple logistic regression analysis, as a function of continuous log_10_ (viral load + 1) and COPD [45]. The contribution of each predictor was analyzed using likelihood ratio tests, with 95% Wald-type CIs computed for the ORs. Duncan et al. documented an adjusted OR of 4.6 (95% CI: 1.2–17.7) in adults ≥ 18 YOA, which was associated with a higher likelihood that an RSV-infected person with COPD would require hospitalization versus an individual without a COPD diagnosis (*p* = 0.02) [45].

#### RSV-Related Complications among Adults with COPD

Seven studies reported on complications among adults with RSV and COPD, including COPD exacerbation [25, 59, 66, 69], ICU admission [61], need for mechanical ventilation [61, 71], and severe clinical outcomes (including ICU admission, mechanical ventilation, and/or RSV-associated death during hospitalization) [46] (Table 2). High percentages of patients suffered exacerbation of COPD (56.8%, 80.4%, and ≥ 83.0%) [25, 59, 69]; however, one study conducted among adults > 40 YOA did not find a statistically significant increase in the risk of acute exacerbation of COPD within 21 days following infection of upper respiratory tract by RSV-A (hazard ratio and 95% CI: 1.2 [0.2–8.8]) or RSV-B (hazard ratio and 95% CI: 1.9 [0.4–8.9]) compared to individuals not infected with RSV [66]. Among inpatients with RSV and COPD, 17.9% were admitted to ICU [61], 9.0–21.1% required mechanical ventilation [61, 71], and 26.4% had severe clinical outcomes [46].

#### Mortality Associated with RSV among Adults with COPD

Three studies documented case fatality rates related to RSV infection among hospitalized adults ≥ 18 YOA with COPD [47, 61, 71], reporting values ranging from 2.8% [61] to 17.8% [71] (Table 3). However, the place and timing for recording mortality differed between studies.

## Discussion

This SLR summarized and quantified published data on the burden of RSV in adults ≥ 18 YOA with asthma or COPD. A substantial number of publications eligible for inclusion were identified, and the methodological quality of most of them was assessed as sufficient according to JBI score. Retrieved publications covered a wide geographical diversity of high-income countries as well as different age groups and patient settings (despite a large focus on inpatient settings). Most included studies reported a relatively high prevalence of asthma and COPD among RSV-infected adults, especially in inpatient settings, suggesting that patients with these respiratory conditions were likely to seek medical care and/or be hospitalized following RSV infection. This SLR also evidenced a significant RSV disease burden in terms of severe complications (exacerbation of underlying respiratory disease, need for mechanical ventilation, admission to ICU) and mortality among adults with asthma or COPD.

Globally, the prevalence of asthma and COPD is country- and age-specific, with recent estimates of 4.3% in adults 18–45 YOA for physician-diagnosed asthma and 10.3% in those 30–79 YOA for COPD [74, 75]. While COPD is increasingly prevalent with age (especially among people ≥ 50 YOA), the overall prevalence of asthma is substantial in both children and adults [74, 76–78]. The present SLR adds further support to the literature suggesting an association between these chronic respiratory conditions and increased propensity for medically attended severe RSV infection [6, 8, 17, 79]. In this regard, the results of this SLR are in line with other studies reporting a disproportionately high prevalence of asthma or COPD among adults with confirmed RSV infection presenting for medical care [17, 79]. For instance, a study from the Centers for Disease Control and Prevention (CDC) showed that asthma and COPD were more prevalent among US adults hospitalized following RSV infection than in the general population, based on a comparison of epidemiological data from the RSV Hospitalization Surveillance Network and the National Center for Health Statistics [80]. Compared to the general population, asthma prevalence was 3.1-fold higher in adults 50–64 YOA with RSV (28.6% versus 9.1%), and 2.2-fold higher in adults ≥ 65 YOA with RSV (17.6% versus 8.0%). For COPD, prevalence was 5.7-fold higher in adults 50–64 YOA with RSV (35.4% versus 6.2%), and 3.4-fold higher in adults ≥ 65 YOA with RSV (33.2% versus 9.8%), compared to the general population [79]. In a broader context, a robust body of evidence emphasized the relatively high prevalence of COPD in hospitalized RSV-infected patients when compared to other respiratory diseases like influenza or COVID-19 [30, 37, 81, 82].

This SLR reported on an increased risk of RSV-related hospitalization for patients with asthma or COPD, which was consistently observed over different age strata. Among all ages, the magnitude of the increase in hospitalization risk was up to 8.2 for asthma and up to 13.4 for COPD. Hence, patients with asthma or COPD consistently had a significantly higher risk of being hospitalized due to more severe RSV infection than adults of the same age without these respiratory comorbidities, even in the lower age groups. These findings corroborate the conclusions derived from another SLR relying on a similar dataset of selected studies (2012–2022 publication period, not exclusively covering respiratory comorbidities), which identified asthma and COPD as significant contributing factors for an elevated risk of hospitalization in RSV-infected adults, independent of age [6]. Likewise, a recent modeling study using national hospital and virologic databases from 2 European countries showed that the risk of RSV hospitalization increased by 1.5- to 3.2-fold in adults ≥ 45 YOA with asthma and by 4.5- to 5.9-fold in those with COPD, compared with the general population [83]. In patients with asthma, hospitalization rates (per 1,000 persons) ranged from 0.7 to 1.6 in adults 45–54 YOA to 47.9–49.5 in those ≥ 85 YOA. In patients with COPD, these rates ranged from 1.6 in people 45–54 YOA to 17.6–39.2 in those ≥ 85 YOA [83].

Several studies included in this SLR showed the high percentage of RSV-related complications in adults with asthma or COPD, among whom the exacerbation of these respiratory conditions was reported as the most frequent. Other significant complications included ICU admission and receipt of mechanical ventilation. This is in agreement with previous studies, which have identified RSV as a major promotor of acute asthma and COPD exacerbations [7, 18–22]. In a prospective cohort of hospitalized adults ≥ 65 YOA and any adult ≥ 18 YOA with underlying cardiopulmonary diseases, RSV infection accounted for 7.2% of hospitalizations for asthma and 11.4% of hospitalizations for COPD [16]. An SLR [19] and a meta-regression analysis [20] focusing on worldwide children and adult asthmatic populations both concluded that RSV was among the most common respiratory pathogens involved in virus-induced asthma exacerbations, with a prevalence of 12.6–13.6% among all age groups and 4.7–5.7% in adults [19, 20]. Similarly, an SLR reported that RSV was diagnosed in 9.9% of adult patients presenting with acute exacerbation of COPD [21], matching a more recent prevalence estimate (8.7%) found in a cohort of outpatient adults ≥ 40 YOA in the United Kingdom [22].

The major contribution of RSV infection to the worsening of asthma or COPD status raises the question about the associated long-term effects on these underlying respiratory diseases and consequences in terms of clinical outcomes, which was not addressed in the present study. Some studies suggest that RSV infection may lead to further deterioration of asthma and COPD, with a permanent negative impact on lung function [27, 84]. For instance, asthmatic patients were found to suffer from a 29% mean maximum reduction in lung function from baseline following RSV respiratory tract infection [84]. In patients with COPD, persistent RSV infection was also associated with an accelerated decline in lung function, independently from exacerbation frequency [27]. Further research is warranted to identify the long-term clinical significance of RSV infection in patients with asthma or COPD, which may extend beyond immediate morbidity.

The strengths of this study include its extensive search strategy, well-defined eligibility criteria, robust data extraction, and quality control measures. In addition, the focus on studies including RSV case definitions relying majorly on PCR diagnosis ensured a relatively high sensitivity and accuracy in detection of RSV-positive cases. However, RSV remains underdiagnosed in adults and diagnostic yield could be further improved with paired serologic analysis to avoid case under-ascertainment arising from PCR testing alone [85–87].

This SLR also has several limitations. First, possible publication bias cannot be excluded. Studies with smaller sample sizes and those with negative findings might not have been published or identified in the search, potentially skewing the overall pooled estimates. Also, some relevant studies may have been missed due to eligibility criteria (e.g., those identifying RSV cases by using ICD codes without statistical adjustments to account for under-detection due to limited testing, those not published in English). Formal statistical tests for publication bias were not included in this analysis. Second, the impact of certain demographic variables like gender and ethnicity on RSV burden in adults with asthma or COPD was not analyzed. This is because the original studies did not provide such stratification, which highlights a limitation in the existing literature. Subgroup estimates (e.g., by comorbidity group or patient setting) were based on independent analyses of included studies rather than model-based approaches. The lack of directly stratified data from individual studies precluded a more nuanced model-based analysis, which could have provided deeper insights into potential associations between RSV infection and asthma- and COPD-related outcomes. Third, methodological quality was evaluated based on the ability of each study to fulfill its own objectives, which may differ from the objectives presented in this SLR. For instance, while the majority of studies included baseline demographic data about the prevalence of asthma or COPD among adults with RSV, this outcome was not part of the main objectives described in these studies. Fourth, there are some limitations inherent to the studies considered in this SLR, first and foremost a high between-study heterogeneity possibly explained by differences in age group classification, settings, participants, severity of RSV disease, RSV diagnosis methods, and definitions of comorbidities. Studies also differed in the length of follow-up period for recording complication and mortality outcomes, which could limit comparability. Some studies had a small sample size (e.g., single-center settings) and may not be representative of the wider population of adults with RSV and asthma or COPD. While heterogeneity was assessed using the *I*² statistic and Cochran’s *Q*-test, these statistical measures cannot fully capture the complexity of differences across studies. This variability may affect the robustness and generalizability of pooled estimates, requiring careful interpretation of the findings. The body of this SLR was also biased toward studies in inpatient settings and severe RSV infections, as few prospective studies were conducted in community settings. A current data gap remains medically attended outpatient RSV disease. This SLR focused on high-income countries and its conclusions may therefore not necessarily extrapolate to low-to-middle-income settings, for which further research is warranted given the paucity of epidemiologic data. Additionally, it is important to acknowledge that some degree of heterogeneity exists in terms of healthcare access and utilization within countries, which could potentially impact the generalizability and interpretation of the present results about asthma and COPD prevalence among adults with RSV. Finally, although sensitivity analyses were conducted for US-based studies, these analyses may not have fully accounted for all sources of heterogeneity and potential unmeasured confounders (e.g., lack of granularity while stratifying by age groups).

There is an increasing number of RSV vaccine recommendations from public health authorities and medical societies, and several note that patients with respiratory conditions like asthma or COPD are among those most likely to benefit from RSV vaccination, including the CDC Advisory Committee on Immunization Practices recommendations [88], the Global Initiative for Asthma (GINA) [89] and the Global Initiative for Chronic Obstructive Lung Disease (GOLD) international guidelines [90]. As of April 27, 2024, a combined total of 10.6 million RSV vaccinations were administered to US adults ≥ 60 YOA in retail pharmacies and physician medical offices [91]. A focus on considerations for RSV vaccination on adults at substantially increased risk of severe RSV disease (e.g., 60–74 YOA with risk factors and all persons ≥ 75 YOA [88]) is warranted and likely to have public health benefit. In this regard, prevention of RSV infection in adults with asthma or COPD through vaccination, consistent with expanded indication for RSV vaccination including adults 50–59 YOA with comorbidities [14], represents a new opportunity to address an important unmet need and improve preventive care.

## Conclusion

Building upon a robust body of evidence gathered between 2000 and 2023, this SLR concludes that adults with asthma or COPD are at increased risk of contracting RSV-induced illness that may lead to hospitalization, various respiratory and general complications (e.g., exacerbation of asthma or COPD, ICU admission, mechanical ventilation), and mortality. In this regard, more research is needed to investigate the association between RSV and severe outcomes in patients with asthma or COPD, especially over the long term. This SLR expands the current body of knowledge in the field of RSV epidemiology in adults ≥ 18 YOA with underlying respiratory conditions by including a wide publication period with updated evidence and an in-depth look at asthma or COPD specifically.

## Electronic Supplementary Material

Below is the link to the electronic supplementary material.

Supplementary Material 1

## Data Availability

No datasets were generated or analysed during the current study.

## Declarations

### Trademark Statement

*Arexvy* is a trademark owned by or licensed to GSK. *Abrysvo* is a trademark of Pfizer Inc. *mRESVIA* is a trademark of Moderna.
