# Supplementary material for: Burden of Respiratory Syncytial Virus Disease in Adults with Asthma and Chronic Obstructive Pulmonary Disease: A Systematic Literature Review
Source: Curr Allergy Asthma Rep. 2025 Feb 25;25(1):14. doi: 10.1007/s11882-025-01194-w (PMC11850418; doi:10.1007/s11882-025-01194-w)
Supplement: Supplementary file 1 — Supplementary Material 1 [file 11882_2025_1194_MOESM1_ESM.docx]

# **Title page**

Burden of respiratory syncytial virus disease in adults with asthma and chronic obstructive pulmonary disease: a systematic literature review

**Authors:** Yolanda Penders^1^, Guy Brusselle^2^, Ann R. Falsey^3^, Gernot Rohde^4^, Estefania Betancur^5^, Maria Elena Guardado^5^, Juan Luis Ramirez Agudelo^5^, Pouya Saeedi^1^, Lauriane Harrington^1^, Jean-Philippe Michaud^1^

**Affiliations:**

^1^ GSK, 1300 Wavre, Belgium

^2^ Department of Respiratory Medicine, Ghent University Hospital, 9000 Ghent, Belgium

^3^ University of Rochester School of Medicine, Rochester, 14642 NY, United States

^4^ Department of Respiratory Medicine, Medical Clinic I, Goethe University Frankfurt, University Hospital, 60590 Frankfurt/Main, Germany

^5^ P95, 3000 Leuven, Belgium

**Corresponding author:**

Name, title: Yolanda Penders

Postal address: GSK, Av. Fleming 20, 1300 Wavre, Belgium

Email: yolanda.x.penders@gsk.com

**Target journal:** Current Allergy and Asthma Reports

**Table of Contents**

[**Title page** 1](#_Toc175061454)

[**Supplementary Information** 3](#_Toc175061455)

[**Online Resource 1** List of inclusion and exclusion criteria 3](#_Toc175061456)

[**Online Resource 2** Detailed search strategy 6](#_Toc175061457)

[**Online Resource 3** List of grey literature sources 10](#_Toc175061458)

[**Online Resource 4** List of publications included 12](#_Toc175061459)

[**References** 18](#_Toc175061460)

# **Supplementary Information**

## **Online Resource 1** List of inclusion and exclusion criteria

| **Domain** | **Inclusion criteria** | **Exclusion criteria** |
| --- | --- | --- |
| **Population** | - Outpatient adults (≥18 YOA) from the general population with laboratory-confirmed RSV infection - Adults (≥18 YOA) from the general population hospitalized with laboratory-confirmed RSV infection - Adults (≥18 YOA) hospitalized with laboratory-confirmed RSV infection and asthma or COPD | - Individuals <18 YOA |
| **Intervention/disease** | - Laboratory-confirmed RSV infection in adults from the general population or with asthma or COPD | - Non-representative (e.g., convenience sample [including non-systematic RSV testing], studies where only influenza-negative patients were tested for other pathogens) or non-relevant population (e.g., immunosuppressed patients such as transplant recipients) - No description of laboratory detection method used to identify RSV-positive cases, or use of rapid antigen testing as the only or the primary diagnostic method to identify most RSV-positive cases - Pooled polymerase chain reaction testing to identify RSV-positive cases and estimate prevalence without testing individual samples in positive pools - Identification of RSV-positive cases using International Classification of Diseases codes without statistical adjustments to account for possible under-detection due to limited testing |
| **Comparison** | - RSV-positive adults with or without asthma or COPD - Results stratified by age group (when available) | - Any other comorbidity |
| **Outcomes** | - Prevalence of asthma or COPD among adults with RSV (%) - Prevalence of RSV-confirmed infections among adults with asthma or COPD (%) - Hospitalization rate associated with RSV infection (per unit of person-time or per population), population count of hospitalizations due to RSV infection, and percentage of hospitalizations among RSV-positive adults with asthma or COPD (%) - Associated risk of hospitalization due to RSV infection among adults with asthma or COPD (incidence rate ratio or odds ratio, crude or adjusted) - Percentage of general or respiratory complications^a^ attributable to RSV infection among adults with asthma or COPD (%) - Case fatality rate associated with RSV infection or following RSV-related hospitalization among adults with asthma or COPD (%) | - Sample size n <20 - Insufficient methodological quality - Missing information in methods/results section to extract relevant data (e.g., numerator or sample size, age of the study population) |
| **Time** | - Studies published between January 1, 2000 and November 28, 2023 |  |
| **Study design** | - Observational studies (case-control, prospective and retrospective cohort, cross-sectional, surveillance) and RCTs^b^ | - SLRs^c^ - Modeling studies - News and opinion articles - Case reports - Narrative reviews, letters/editorials, or comments - Genetic or molecular studies (including studies conducted in animal and human cellular models) - Studies on RSV vaccination during pregnancy |
| **Others** | - Articles published in English - High-income countries (based on World Bank country classification by income level)^d^ |  |

^a^ General complications included admission to intensive care unit, length of hospital stay, emergency room visits, and deterioration in functional status. Respiratory complications included exacerbation of asthma or COPD (also considering severity as defined in the study), pneumonia, respiratory co-infection, need for mechanical ventilation, and additional therapies (short-acting beta agonists for asthma, oral corticosteroids, antibiotics, antivirals).

^b^ Information from different treatment arms in an RCT were extracted provided that all eligibility criteria were met (e.g., for trials with an antiviral medication, either the treatment or placebo/usual care arm needed to meet the sample size criteria of n ≥20 separately).

^c^ SLRs covering the objectives were included during the title and abstract screening phase to identify additional relevant studies within the reference lists.

^d^ The following countries were included in geographical scope: American Samoa, Andorra, Antigua and Barbuda, Aruba, Australia, Austria, Bahrain, Barbados, Belgium, Bermuda, British Virgin Islands, Brunei Darussalam, Canada, Cayman Islands, Channel Islands, Chile, Croatia, Curaçao, Cyprus, Czech Republic, Denmark, Estonia, Faroe Islands, Finland, France, French Polynesia, Guyana, Germany, Gibraltar, Greece, Greenland, Guam, Hong Kong Special Administrative Region, Hungary, Iceland, Ireland, Isle of Man, Israel, Italy, Japan, Kuwait, Latvia, Liechtenstein, Lithuania, Luxembourg, Macao Special Administrative Region, Malta, Monaco, Nauru, New Caledonia, New Zealand, Northern Mariana Islands, Norway, Oman, Panama, Poland, Portugal, Puerto Rico, Qatar, Republic of Korea, Romania, Saint Kitts and Nevis, Saint Martin, San Marino, Saudi Arabia, Seychelles, Singapore, Slovak Republic, Slovenia, Spain, Sweden, Switzerland, Taiwan, The Bahamas, The Netherlands, Trinidad and Tobago, Turks and Caicos Islands, United Arab Emirates, United Kingdom, United States, Uruguay, Virgin Islands (United States).

Abbreviations: COPD, chronic obstructive pulmonary disease; n, number of participants; RCT, randomized controlled trial; RSV, respiratory syncytial virus; SLR, systematic literature review; YOA, years of age.

## **Online Resource 2** Detailed search strategy

| **Domain** | **Query** | **Number of hits** |
| --- | --- | --- |
| **PubMed (date of search: November 28, 2023)** | | |
| **10. Update** | (#1 AND (#2 OR #3 OR #4) NOT (#5 OR #6 OR #7)) Filters: Abstract, English, from 2023/9/1–2023/11/28 | **286** |
| **9. Filters** | (#1 AND (#2 OR #3 OR #4) NOT (#5 OR #6 OR #7)) Filters: Abstract, English, from 2000/1/1–2023/11/28 | 9,314 |
| **8. Combined search** | (#1 AND (#2 OR #3 OR #4) NOT (#5 OR #6 OR #7)) | 11,645 |
| **7. Inadequate abbreviated terms** | resveratrol[tiab] OR Rous sarcoma virus[tiab] OR Relative search volume[tiab] OR Right subclinical varicocele[tiab] OR Rosuvastatin[tiab] OR Ratio subcutaneous to visceral fat[tiab] OR Rice stripe virus[tiab] OR Recurrent spontaneous vertigo[tiab] OR Retinal slip velocity[tiab] OR Right sinus of valsalva[tiab] OR Mean platelet volume[tiab] | 31,275 |
| **6. Non-pertinent publication types** | case reports[pt] OR editorial[pt] OR letter[pt] OR news[pt] OR comment[pt] | 4,549,147 |
| **5. Animal studies** | Animals[Mesh] NOT (Humans[Mesh] AND Animals[Mesh]) | 5,172,009 |
| **4. Risk factors** | caus*[tiab] OR predictor*[tiab] OR determinant*[tiab] OR risk factor*[tiab] OR correlation*[tiab] OR origin*[tiab] OR underlying[tiab] | 6,850,163 |
| **3. Complications** | “Morbidity”[Mesh] OR morbidity[tiab] OR complication*[tiab] OR "Mortality"[Mesh] OR “Mortality”[subheading] OR mortality[tiab] OR mortalities[tiab] OR "Death"[Mesh] OR death*[tiab] OR case-fatalit*[tiab] OR lethal*[tiab] OR died[tiab] OR "Hospitalisation"[Mesh] OR hospital*[tiab] OR "General Practitioners"[Mesh] OR general practitioner[tiab] OR general practitioners[tiab] OR GP[tiab] OR “Delivery of Health Care"[Mesh] OR health care[tiab] OR healthcare[tiab] OR "Ambulatory Care"[Mesh] OR ambulator*[tiab] OR primary care [tiab] OR health resource*[tiab] OR "Critical Care"[Mesh] OR critical care[tiab] OR intensive care[tiab] OR ICU[tiab] OR "Inpatients"[Mesh] OR inpatient*[tiab] OR visit*[tiab] OR consultation*[tiab] OR emergency room[tiab] OR ER[tiab] OR emergency department[tiab] OR ED[tiab] | 7,102,053 |
| **2. Measures of frequency** | "Incidence"[Mesh] OR inciden*[tiab] OR "Prevalence"[Mesh] OR prevalen*[tiab] OR frequency[tiab] OR frequencies[tiab] OR rate*[tiab] OR proportion*[tiab] OR distribut*[tiab] | 7,401,728 |
| **1. RSV** | "Respiratory Syncytial Viruses"[MeSH] OR "Respiratory Syncytial Virus Infections"[Mesh] OR respiratory syncytial[tiab] OR RSV[tiab] | 23,893 |
| **Embase (date of search: November 28, 2023)** | | |
| **10. Update** | #1 AND (#2 OR #3 OR #4) AND #6 NOT (#5 OR #7) AND [english]/lim AND [abstracts]/lim AND [01-09-2023]/sd NOT [29-11-2023]/sd | **405** |
| **9. Filters** | #1 AND (#2 OR #3 OR #4) AND #6 NOT (#5 OR #7) AND [english]/lim AND [abstracts]/lim AND [01-01-2000]/sd NOT [28-11-2023]/sd | 12,616 |
| **8. Combined search** | #1 AND (#2 OR #3 OR #4) AND #6 NOT (#5 OR #7) | 15,688 |
| **7. Inadequate abbreviated terms** | resveratrol:ti,ab OR Rous sarcoma virus:ti,ab OR Relative search volume:ti,ab OR Right subclinical varicocele:ti,ab OR Rosuvastatin:ti,ab OR Ratio subcutaneous visceral fat:ti,ab OR Rice stripe virus:ti,ab OR Recurrent spontaneous vertigo:ti,ab OR Retinal slip velocity:ti,ab OR Right sinus of valsalva:ti,ab OR Mean platelet volume:ti,ab | 11,666 |
| **6. Pertinent publication types** | [article]/lim OR [article in press]/lim OR [review]/lim | 33,083,644 |
| **5. Animal studies** | 'animal'/exp NOT ('human'/exp AND 'animal'/exp) | 6,047,272 |
| **4. Risk factors** | caus*:ti,ab OR predictor*:ti,ab OR determinant*:ti,ab OR “risk factor*”:ti,ab OR correlation*:ti,ab OR origin*:ti,ab OR underlying:ti,ab | 8,993,706 |
| **3. Complications** | 'morbidity'/exp OR morbidity:ti,ab OR complication*:ti,ab OR 'mortality'/exp OR 'mortality':lnk OR mortality:ti,ab OR mortalities:ti,ab OR 'death'/exp OR death*:ti,ab OR “case-fatalit*”:ti,ab OR lethal*:ti,ab OR died:ti,ab OR 'hospitalisation'/exp OR hospital*:ti,ab OR 'general practitioner'/exp OR “general practitioner”:ti:ab OR “general practitioners”:ti:ab OR GP:ti,ab OR ‘Health care delivery’/exp OR “health care”:ti:ab OR healthcare:ti,ab OR 'ambulatory care'/exp OR ambulator*:ti,ab OR “primary care clinic”:ti,ab OR “health resource*”:ti,ab OR ‘Intensive Care’/exp OR “intensive care”:ti:ab OR “critical care”:ti,ab OR ICU:ti:ab OR 'hospital patient'/exp OR inpatient*:ti,ab OR visit*:ti,ab OR consultation*:ti,ab OR “emergency room”:ti,ab OR ER:ti,ab OR “emergency department”:ti,ab OR ED:ti,ab | 10,635,764 |
| **2. Measures of frequency** | 'incidence'/exp OR inciden*:ti,ab OR 'prevalence'/exp OR prevalen*:ti,ab OR frequency:ti,ab OR frequencies:ti,ab OR rate*:ti,ab OR proportion*:ti,ab OR distribut*:ti,ab | 9,975,801 |
| **1. RSV** | 'Human respiratory syncytial virus'/exp OR 'respiratory syncytial virus infection'/exp OR “respiratory syncytial”:ti,ab OR RSV:ti,ab | 35,878 |

Abbreviation: Mesh, Medical Subject Headings; RSV, respiratory syncytial virus.

Note: [tiab]/ti.ab/ti,ab/ti:ab search-related terms indicate that a specific term was searched only in title/abstract.

## **Online Resource 3** List of grey literature sources

| **Source** | **Search terms** | **Search strategy** | **Number of hits** |
| --- | --- | --- | --- |
| **CDC – RSV working group presentations [1]** | NA | - Website (RSV page – RSV in older adults and adults with chronic medical conditions) - Slides from the Advisory Committee on Immunisation Practices work groups in RSV Vaccines – Adult | NA |
| **ReSViNET – RSVVW conference abstracts [2]** | NA | - Website (conference abstracts from 2023) | NA |
| **IDWeek abstracts [3]** | “RSV’’ OR “respiratory syncytial virus’’ | - Oral and poster abstracts in Open Forum Infectious Diseases (from 2021 and 2022) | NA |
| **ATS [4]** | “RSV’’ OR “respiratory syncytial virus’’ | - Website (conference abstracts from San Francisco, 2022 and Washington, 2023) | 55 |
| **ERS [5]** | “RSV’’ OR “respiratory syncytial virus’’^a^ AND “comorbidities’’ | - Screening of the first 10 pages - Screening of ERS website (Respiratory infections – Publications) | 558 |
| **EAACI [6]** | “respiratory syncytial virus’’ | - Website (knowledge hub for professionals) - EAACI 2022 presentations | NA |
| **AAAAI [7]** | “respiratory syncytial virus’’ | - Latest research summaries – The Journal of Allergy and Clinical Immunology (from 2022 and 2023) - Conditions Library - search results - Asthma articles | 53 |
| **CHEST [8]** | “RSV’’ OR “respiratory syncytial virus’’^a^ AND “comorbidities” | - Website (Respiratory syncytial virus (RSV): measuring the impact and vaccine prevention) - CHEST journal | 96 |
| **ECCMID [9]** | “RSV’’ OR “respiratory syncytial virus’’ | - Posters and poster sessions from 2022 and 2023 – Viral infection & disease (non-COVID-19) – Influenza and respiratory viruses - ESCMID library | NA |
| **RSV symposium[10]** | NA | - 12^th^ IRSVS 2022 – Abstracts: posters | NA |

^a^ “respiratory syncytial virus” was also considered as an additional, separate search term for this database.

Abbreviations: AAAAI, American Academy of Allergy, Asthma & Immunology; ATS, American Thoracic Society; CDC, Centers for Disease Control and Prevention; CHEST, American College of Chest Physicians; COVID-19, coronavirus disease 2019; EAACI, European Academy of Allergy & Clinical Immunology; ECCMID, European Conference of Clinical Microbiology and Infectious Diseases; ESCMID, European Society of Clinical Microbiology and Infectious Diseases; ERS, European Respiratory Society; IDWeek, Infectious Diseases Week; IRSVS, International Respiratory Syncytial Virus Society; NA, not applicable; ReSViNET, Respiratory Syncytial Virus Foundation; RSV, respiratory syncytial virus.

## **Online Resource 4** List of publications included

| **Reference**  **(study location)** | **Study design** | **Period** | **Patient settings** | **Total sample size** | **Gender, female (%)** | **Age in years (mean ± SD or median [IQR])** | **Quality^a^** | **Asthma** | **COPD** |
| --- | --- | --- | --- | --- | --- | --- | --- | --- | --- |
| **North America** | | | | | | | | | |
| **Ackerson, 2019^b^ [11]**  **(US)** | Retrospective cohort | 2011–2015 | I | 645 | 60.5 | 78.5 ± 9.8 | Sufficient | X | X |
| **Ackerson, 2020^b^ [12]**  **(US)** | Retrospective cohort | 2011–2015 | I | 579 | 58.5 | 78.4 ± 9.8 | Sufficient | X | X |
| **Barrett, 2020 [13]**  **(US)** | Retrospective cohort (outbreak study) | 2019 (1 month) | O (LTCF) | 42 | 7.1 | 73 [47–89] | Poor |  | X |
| **Belongia, 2018^c^ [14]**  **(US)** | Retrospective cohort | 2004–2005 to 2015–2016 | M(I/O) | 2,257 | 60.5 | NR | Poor | X | X |
| **Binder, 2017 [15]**  **(US)** | Retrospective cohort | 2007–2014 | I | 295 | 47 | Mean: 66.5 (range: 19–97) | Poor |  | X |
| **Branche, 2022 [16]**  **(US)** | Prospective, population-based, active surveillance | 2017–2020 | I | 1,039 | 60 | 69 [58–81] | Sufficient | X | X |
| **Branche, 2022 (2)^d^ [17]**  **(US)** | Prospective, population-based, active surveillance | 2017–2020 | I | 302 | 63 | 74 [67–82] | Sufficient | X | X |
| **Duncan, 2009 [18]**  **(US)** | Prospective, cross-sectional | 2007–2008 | I; O | I: 32  O: 26 | I: 34 O:58 | I: 71 ± 13 O: 65 ± 14 | Sufficient | X | X |
| **Goldman, 2022 [19]**  **(US)** | Prospective, population-based, active surveillance | 2017–2018, 2018–2019 | I | 403 | 58.3 | 69 [57.2–82.1] | Sufficient | X | X |
| **Havers, 2023 [20]**  **(US)** | Population-based, surveillance | 2022–2023 | I | 3,218 | 60.5 | 75 [68–84] | Sufficient | X | X |
| **Juhn, 2023 [21]**  **(US)** | Prospective cohort | 2019–2021 | O | 2,325 | 59.4 | 67.7 ± 10.0 | Sufficient | X | X |
| **Khurana, 2023^e^ [22]**  **(US)** | Retrospective cohort | 2010–2017 | I | 856 | 60 | 70 ± 17.5 | Sufficient |  | X |
| **Kujawski, 2022 [23]**  **(US)** | Retrospective cohort | 2015–2017 | I | 2,042 | 59.2 | 69 [57–82] | Sufficient | X | X |
| **Lee, 2011 [24]**  **(US)** | Prospective cohort | 2005–2008 | I | 50 | Steroid treatment: 30  Non-steroid treatment: 53 | Steroid treatment: 69.8 ± 14.9  Non-steroid treatment: 72 ± 14.8 | Poor | X | X |
| **Lee, 2019 [25]**  **(US)** | Retrospective cohort review analysis of individual patient data | 2014–2016 | I | 379 | 44.4 | 60 ± 16 | Sufficient | X | X |
| **Malosh, 2017 [26]**  **(US)** | Prospective cohort | 2014–2015, 2015–2016 | I | 1,259 | NR | NR | Sufficient |  | X |
| **McClure, 2014^c^ [27]**  **(US)** | Prospective cohort | 2006–2007 to 2009–2010 | M(I/O) | 20,453 | 53 | 63.7 ± 12.1 | Sufficient |  | X |
| **Mehta, 2013 [28]**  **(US)** | Post-hoc analyses of 2 prospective cohorts | 1999–2003 and 2004 (2 months) | M(I/O) | 379 | 51.2 | 69.9 ± 9.6 | Sufficient |  | X |
| **Mulpuru, 2022 [29]**  **(Canada)** | Prospective cohort | 2011–2015 | I | 3,931 | Positive NP swab: 50 Negative NP swab: 48.8 | Positive NP swab: 73 ± 12.4 Negative NP swab: 73 ± 11.3 | Sufficient |  | X |
| **Sano, 2022 [30]**  **(US)** | Retrospective cohort, surveillance-based | 2017–2018, 2018–2019 | I | 365 | 61.3 | BC collected: 69 ± 18  No BC collected: 68 ± 19 | Sufficient | X | X |
| **Smithgall, 2020 [31]**  **(US)** | Retrospective cohort, surveillance-based | 2013–2015 | I | 1,102 | 50.2 | NR | Sufficient | X | X |
| **Sundaram, 2014 [32]**  **(US)** | Retrospective cohort | 2004–2010 | M(I/O) | 2,225 | 60.8 | 64.3 ± 10.7 | Sufficient |  | X |
| **Tseng, 2020 [33]**  **(US)** | Retrospective cohort | 2011–2015 | I | 664 | 60.5 | 78 [60–103] | Sufficient | X | X |
| **Walsh, 2013 [34]**  **(US)** | Prospective cohort | 2005–2008 | I; O | I: 50  O: 61 | NR | I: 70.5 ± 14.7  O: 56.7 ± 17.1 | Sufficient |  | X |
| **Europe** | | | | | | | | | |
| **Ambrosch, 2023 [35]**  **(Germany)** | Retrospective cohort | 2017–2020 | I | 318 | 42.5 | 75.1 ± 14.3 | Poor |  | X |
| **Chorazka, 2021^f^ [36]**  **(Switzerland)** | Retrospective cohort | 2017–2019 | I | 1,983 | 60.8 | 78 [65–84] | Sufficient | X | X |
| **Coussement, 2022 [37]**  **(France, Belgium)** | Retrospective cohort | 2011–2018 | I | 618 | 45.3 | 67.2 ± 15.0 | Sufficient | X | X |
| **Debes, 2022 [38]**  **(Norway)** | Retrospective cohort | 2015–2018 | I | 1,182 | 53 | 69.6 ± 16.1 | Sufficient |  | X |
| **Hämäläinen, 2022^f^ [39]**  **(Finland)** | Retrospective cohort | 2017–2018 | I | 725 | 50.7 | 73.3 ± 15.1 | Sufficient | X | X |
| **Korsten, 2021 [40]**  **(Belgium, The Netherlands, United Kingdom)** | Prospective cohort | 2017–2019 | O | 1,040 | 54 | Median: 75 | Sufficient | X | X |
| **Losa-Martin, 2023 [41]**  **(Spain)** | Retrospective cohort | 2018–2019 | I | 284 | 52.3 | 71.6 ± 15.9 | Sufficient | X | X |
| **Santus, 2023 [42]**  **(Italy)** | Retrospective cohort | 2022–2023 | M(I/O) | 717 | 47.2 | 80 [71–82] | Sufficient | X | X |
| **Stolz, 2019 [43]**  **(Switzerland)** | Randomized controlled trial | 2011–2015 | O | 445 | 32.6 | 66.9 ± 9.4 | Sufficient |  | X |
| **Subissi, 2020 [44]**  **(Belgium)** | Retrospective cohort, surveillance-based | 2018–2019 | I | 508 | 44.9 | NR | Sufficient | X |  |
| **Asia** | | | | | | | | | |
| **Kwon, 2017^f^ [45]**  **(Republic of Korea)** | Retrospective cohort | 2013–2015 | I | 339 | 49.9 | 70 ± 12.2 | Sufficient | X | X |
| **Levinson, 2023 [46]**  **(Israel)** | Cross-sectional | 2012–2021 | I | 1,124 | 51 | 76.1 [63.9–85.4] | Sufficient |  | X |
| **Wong, 2014 [47]**  **(Hong Kong)** | Retrospective cohort | 2009–2011 | I | 285 | 48.1 | 74 ± 16 | Sufficient | X | X |
| **Oceania** | | | | | | | | | |
| **Minney-Smith, 2019 [48]**  **(Australia)** | Retrospective cohort | 2012–2015 | I | 1,753 | 48.2 | NR | Sufficient | X | X |
| **Prasad, 2021 [49]**  **(New Zealand)** | Surveillance-based | 2012–2015 | I | 883,999 | NR | NR | Sufficient | X | X |
| **South America** | | | | | | | | | |
| **Luchsinger, 2012 [50]**  **(Chile)** | Prospective cohort | 2005–2007 | M(I/O) | 356 | 46.6 | Mean: 63 | Sufficient | X | X |

^a^ The methodological quality of each study was assessed using the quality appraisal tools developed by the Joanna Briggs Institute at the University of Adelaide, Australia [51].

^b^ Ackerson, 2019 and Ackerson, 2020 were excluded from the analysis of pooled prevalence estimates due to data overlapping with Tseng, 2020.

^c^ Belongia, 2018 and McClure, 2014 were excluded from the analysis of pooled prevalence estimates due to data overlapping with Sundaram, 2014.

^d^ Branche, 2022 (2) was excluded from the analysis of pooled prevalence estimates due to data overlapping with Branche, 2022.

^e^ Abstract.

^f^ Gender and age data are reported for RSV subpopulation.

Abbreviations: BC, blood culture; COPD, chronic obstructive pulmonary disease; I, inpatient; IQR, interquartile range; LTCF, long-term care facilities; M(I/O), mixed inpatient/outpatient; NP, nasopharyngeal; NR, not reported; O, outpatient; RSV, respiratory syncytial virus; SD, standard deviation; US, United States.

# **References**

1. CDC. About RSV. <https://www.cdc.gov/rsv/index.html>. Accessed 21 Aug 2024.

2. ReSViNET. Reduce the global burden of RSV infection. 2024. <https://resvinet.org/>. Accessed 21 Aug 2024.

3. IDWeek. 2024. <https://idweek.org/>. Accessed 21 Aug 2024.

4. ATS. 2024. <https://www.thoracic.org/>. Accessed 21 Aug 2024.

5. ERS. <https://www.ersnet.org/>. Accessed 21 Aug 2024.

6. EAACI. 2023. <https://eaaci.org/>. Accessed 21 Aug 2024.

7. AAAAI. 2024. <https://www.aaaai.org/>. Accessed 21 Aug 2024.

8. CHEST. 2024. <https://www.chestnet.org/>. Accessed 21 Aug 2024.

9. ECCMID. 2024. <https://www.eccmid.org/>. Accessed 21 Aug 2024.

10. EFCNI. 12th International RSV Symposium (RSV2022). 2021. <https://www.efcni.org/event/12th-international-rsv-symposium-rsv2022/>. Accessed 21 Aug 2024.

11. Ackerson B, Tseng HF, Sy LS, Solano Z, Slezak J, Luo Y, et al. Severe morbidity and mortality associated with respiratory syncytial virus versus influenza infection in hospitalized older adults. Clin Infect Dis. 2019;69(2):197–203.

12. Ackerson B, An J, Sy LS, Solano Z, Slezak J, Tseng HF. Cost of hospitalization associated with respiratory syncytial virus infection versus influenza infection in hospitalized older adults. J Infect Dis. 2020;222(6):962–6.

13. Barrett N, Bailey L, Ford F, Thorne M, Azab N, LeMaitre B, et al. Respiratory syncytial virus outbreak in a veterans affairs long-term care facility. Infect Dis Clin Prac. 2020;28(4):200–3.

14. Belongia EA, King JP, Kieke BA, Pluta J, Al-Hilli A, Meece JK, et al. Clinical features, severity, and incidence of RSV illness during 12 consecutive seasons in a community cohort of adults ≥60 years old. Open Forum Infect Dis. 2018;5(12):ofy316.

15. Binder W, Thorsen J, Borczuk P. RSV in adult ED patients: do emergency providers consider RSV as an admission diagnosis? Am J Emerg Med. 2017;35(8):1162–5.

16. Branche AR, Saiman L, Walsh EE, Falsey AR, Sieling WD, Greendyke W, et al. Incidence of respiratory syncytial virus infection among hospitalized adults, 2017–2020. Clin Infect Dis. 2022;74(6):1004–11.

17. Branche AR, Saiman L, Walsh EE, Falsey AR, Jia H, Barrett A, et al. Change in functional status associated with respiratory syncytial virus infection in hospitalized older adults. Influenza Other Respir Viruses. 2022;16(6):1151–60.

18. Duncan CB, Walsh EE, Peterson DR, Lee FEH, Falsey AR. Risk factors for respiratory failure associated with respiratory syncytial virus infection in adults. J Infect Dis. 2009;200(8):1242–6.

19. Goldman CR, Sieling WD, Alba LR, Silverio Francisco RA, Vargas CY, Barrett AE, et al. Severe clinical outcomes among adults hospitalized with respiratory syncytial virus infections, New York City, 2017–2019. Public Health Rep. 2022;137(5):929–35.

20. Havers FP, Whitaker M, Melgar M, Chatwani B, Chai SJ, Alden NB, et al. Characteristics and outcomes among adults aged ≥60 years hospitalized with laboratory-confirmed respiratory syncytial virus - RSV-NET, 12 States, July 2022–June 2023. Am J Transplant. 2023;23(12):2000–7.

21. Juhn YJ, Wi CI, Takahashi PY, Ryu E, King KS, Hickman JA, et al. Incidence of respiratory syncytial virus infection in older adults before and during the COVID-19 pandemic. JAMA Netw Open. 2023;6(1):e2250634.

22. Khurana S, Wallace M, Chandler T, Furmanek S, Ramirez JA, Cavallazzi R. Clinical characteristics and outcomes of respiratory syncytial virus pneumonia in comparison to influenza or pneumococcal pneumonia in adults. Am J Respir Crit Care Med. 2023;207:A6749.

23. Kujawski SA, Whitaker M, Ritchey MD, Reingold AL, Chai SJ, Anderson EJ, et al. Rates of respiratory syncytial virus (RSV)-associated hospitalization among adults with congestive heart failure-United States, 2015–2017. PLoS One. 2022;17(3):e0264890.

24. Lee FEH, Walsh EE, Falsey AR. The effect of steroid use in hospitalized adults with respiratory syncytial virus-related illness. Chest. 2011;140(5):1155–61.

25. Lee N, Walsh EE, Sander I, Stolper R, Zakar J, Wyffels V, et al. Delayed diagnosis of respiratory syncytial virus infections in hospitalized adults: individual patient data, record review analysis and physician survey in the United States. J Infect Dis. 2019;220(6):969–79.

26. Malosh RE, Martin ET, Callear AP, Petrie JG, Lauring AS, Lamerato L, et al. Respiratory syncytial virus hospitalization in middle-aged and older adults. J Clin Virol. 2017;96:37–43.

27. McClure DL, Kieke BA, Sundaram ME, Simpson MD, Meece JK, Sifakis F, et al. Seasonal incidence of medically attended respiratory syncytial virus infection in a community cohort of adults ≥50 years old. PLoS One. 2014;9(7):e102586.

28. Mehta J, Walsh EE, Mahadevia PJ, Falsey AR. Risk factors for respiratory syncytial virus illness among patients with chronic obstructive pulmonary disease. COPD. 2013;10(3):293–9.

29. Mulpuru S, Andrew MK, Ye L, Hatchette T, LeBlanc J, El-Sherif M, et al. Impact of respiratory viral infections on mortality and critical illness among hospitalized patients with chronic obstructive pulmonary disease. Influenza Other Respir Viruses. 2022;16(6):1172–82.

30. Sano E, Chang B, Sieling W, Jay R, Hill-Ricciuti A, Phillips M, et al. Bacteremia in adults admitted from the emergency department with laboratory-confirmed respiratory syncytial virus. J Emerg Med. 2022;62(2):216–23.

31. Smithgall M, Maykowski P, Zachariah P, Oberhardt M, Vargas CY, Reed C, et al. Epidemiology, clinical features, and resource utilization associated with respiratory syncytial virus in the community and hospital. Influenza Other Respir Viruses. 2020;14(3):247–56.

32. Sundaram ME, Meece JK, Sifakis F, Gasser RA, Jr, Belongia EA. Medically attended respiratory syncytial virus infections in adults aged ≥50 years: clinical characteristics and outcomes. Clin Infect Dis. 2014;58(3):342–9.

33. Tseng HF, Sy LS, Ackerson B, Solano Z, Slezak J, Luo Y, et al. Severe morbidity and short- and mid- to long-term mortality in older adults hospitalized with respiratory syncytial virus infection. J Infect Dis. 2020;222(8):1298–310.

34. Walsh EE, Peterson DR, Kalkanoglu AE, Lee FEH, Falsey AR. Viral shedding and immune responses to respiratory syncytial virus infection in older adults. J Infect Dis. 2013;207(9):1424–32.

35. Ambrosch A, Luber D, Klawonn F, Kabesch M. Focusing on severe infections with the respiratory syncytial virus (RSV) in adults: risk factors, symptomatology and clinical course compared to influenza A / B and the original SARS-CoV-2 strain. J Clin Virol. 2023;161:105399.

36. Chorazka M, Flury D, Herzog K, Albrich WC, Vuichard-Gysin D. Clinical outcomes of adults hospitalized for laboratory confirmed respiratory syncytial virus or influenza virus infection. PLoS One. 2021;16(7):e0253161.

37. Coussement J, Zuber B, Garrigues E, Gros A, Vandueren C, Epaillard N, et al. Characteristics and outcomes of patients in the ICU with respiratory syncytial virus compared with those with influenza infection: a multicenter matched cohort study. Chest. 2022;161(6):1475–84.

38. Debes S, Haug JB, de Blasio BF, Lindstrøm JC, Jonassen CM, Dudman SG. Clinical outcome of viral respiratory tract infections in hospitalized adults in Norway: high degree of inflammation and need of emergency care for cases with respiratory syncytial virus. Front Med. 2022;9:866494.

39. Hämäläinen A, Savinainen E, Hämäläinen S, Sivenius K, Kauppinen J, Koivula I, et al. Disease burden caused by respiratory syncytial virus compared with influenza among adults: a retrospective cohort study from Eastern Finland in 2017–2018. BMJ Open. 2022;12(12):e060805.

40. Korsten K, Adriaenssens N, Coenen S, Butler C, Ravanfar B, Rutter H, et al. Burden of respiratory syncytial virus infection in community-dwelling older adults in Europe (RESCEU): an international prospective cohort study. Eur Respir J. 2021;57(4):2002688.

41. Losa-Martin O, Frisuelos-Garcia A, Delgado-Iribarren A, Martin-deCabo MR, Martin-Segarra O, Vegas-Serrano A, et al. Respiratory syncytial virus infection in adults: differences with influenza. Enferm Infecc Microbiol Clin. 2023;42:62–8.

42. Santus P, Radovanovic D, Gismondo MR, Rimoldi SG, Lombardi A, Danzo F, et al. Respiratory syncytial virus burden and risk factors for severe disease in patients presenting to the emergency department with flu-like symptoms or acute respiratory failure. Respir Med. 2023;218:107404.

43. Stolz D, Papakonstantinou E, Grize L, Schilter D, Strobel W, Louis R, et al. Time-course of upper respiratory tract viral infection and COPD exacerbation. Eur Respir J. 2019;54(4):1900407.

44. Subissi L, Bossuyt N, Reynders M, Gérard M, Dauby N, Bourgeois M, et al. Capturing respiratory syncytial virus season in Belgium using the influenza severe acute respiratory infection surveillance network, season 2018/19. Euro Surveill. 2020;25(39):1900627.

45. Kwon YS, Park SH, Kim MA, Kim HJ, Park JS, Lee MY, et al. Risk of mortality associated with respiratory syncytial virus and influenza infection in adults. BMC Infect Dis. 2017;17(1):785.

46. Levinson T, Wasserman A, Shenhar-Tsarfaty S, Halutz O, Shapira I, Zeltser D, et al. Comparative analysis of CRP as a biomarker of the inflammatory response intensity among common viral infections affecting the lungs: COVID-19 versus influenza A, influenza B and respiratory syncytial virus. Clin Exp Med. 2023;23:5307–13.

47. Wong SSM, Yu JWL, Wong KT, Lee N, Lui GCY, Chan PKS, et al. Initial radiographic features as outcome predictor of adult respiratory syncytial virus respiratory tract infection. AJR Am J Roentgenol. 2014;203(2):280–6.

48. Minney-Smith CA, Selvey LA, Levy A, Smith DW. Post-pandemic influenza A/H1N1pdm09 is associated with more severe outcomes than A/H3N2 and other respiratory viruses in adult hospitalisations. Epidemiol Infect. 2019;147:e310.

49. Prasad N, Walker TA, Waite B, Wood T, Trenholme AA, Baker MG, et al. Respiratory syncytial virus-associated hospitalizations among adults with chronic medical conditions. Clin Infect Dis. 2021;73(1):e158–e163.

50. Luchsinger V, Piedra PA, Ruiz M, Zunino E, Martínez MA, Machado C, et al. Role of neutralizing antibodies in adults with community-acquired pneumonia by respiratory syncytial virus. Clin Infect Dis. 2012;54(7):905–12.

51. JBI. Critical Appraisal Tools. <https://jbi.global/critical-appraisal-tools>. Accessed 21 Aug 2024.
